# Molecular and Cellular Mechanisms of Human Astrocytoma Progression: Advances in Knowledge to Reach Therapeutic Horizons

**DOI:** 10.3390/cells9102216

**Published:** 2020-09-30

**Authors:** Sergio Comincini

**Affiliations:** Laboratory of Functional Oncogenomics, Department of Biology and Biotechnology, University of Pavia, 27100 Pavia, Italy; sergio.comicnini@unipv.it; Tel.: +39-038-298-5529; Fax: +39-038-252-8496

**Keywords:** astrocytic tumor, Glioblastoma multiforme, glioma, gene-therapy, gene-interfering, programmed cell death

## Abstract

Human astrocytic tumors are primary central nervous system (CNS) tumors that arise either from astrocytes or from precursor cells. A growing number of epidemiological and incidence studies in different countries underlined that, in addition to increasing economic costs for health systems, these cancers are still representing one of the main hurdles in developing a successful therapeutic goal for patients. On the other hand, new-omics technologies are offering customized instruments and more and more advantageous results toward personalized medicine approaches, underlining the concept that each tumor mass undergoes a peculiar transformation process under the control of specific genes’ and proteins’ functional signatures. The main aim of this Special Issue was to collect novel contributions in the wide field of human tumor astrocytic basic and translational research, to suggest further potential therapeutic targets/strategies that might interfere, possibly at the earliest stage of transformation, with the tumor progression, and to increase the molecular-based arsenal to counteract the prognostic poverty of high-grade astrocytic tumors.

Malignant glial tumors (gliomas and in particular Glioblastoma multiforme, GBM) are, after cerebral stroke, the second cause of death in CNS diseases. The current standard clinical interventions are based on specifically designed combinations of tumor resection, radio-, and chemo-therapy protocols. However, at present, these therapeutic approaches do not result in significant improvement of the average survival of patients with, in the case of the highest malignant GBM, a median survival of 12–15 months. 

Consequently, this Special Issue has collected different multidisciplinary research articles describing novel potential therapeutic strategies against gliomas [1,2], the identification of molecular signatures that might interfere with tumor-associated phenomena as hypoxia and vascularization, transformation and invasion [3,4,5]; in addition, this Issue has selected new contributions related to molecular and cellular characterization of GBM-derived stem cells to shed light on the tumorigenesis and to suggest novel therapeutic strategies for gliomas [6,7,8,9].

In the first contribution, Bilmin and collaborators [1] focused on a specific variant of the sonodynamic therapy (SDT), defined as the application of non-thermal ultrasound energy for the treatment of diseases in combination with drugs. SDT employs a principle similar to photodynamic therapy of superficially located cancers that adopt ultrasound instead of light waves to deliver the energy necessary to eradicate sensitized malignant cells [10]. Importantly, the ability of ultrasound to penetrate brain tissues makes it possible to reach deeply localized intracranial tumors such as gliomas. Furthermore, an important advantage of the SDT approach is its relative non-invasiveness and thus the possibility to schedule repeated applications. In detail, the authors reported in vitro and in vivo studies on the use of 5-aminolevulinic acid as a sonosensitizer molecule to interfere with the tumor cells proliferation in recurring gliomas [11,12]. According to the authors, the illustrated pieces of evidence are encouraging, but further studies will be required to define the optimal experimental parameters of the ultrasonic waves required to induce a more effective sonodynamic response in malignant cells, avoiding significant off-targets effects to the surrounding healthy brain tissues. In conclusion, the described results could be a starting milestone for planning further preclinical in vivo studies, and, in the future, directed to clinical trials of gliomas.

In a different contribution, always on the therapeutic side, Guidotti and co-workers [2] dissect the use of different peptides as promising anticancer agents in gliomas. The authors, in particular, highlight different studies on the efficacy of peptides to promote the reduction of tumor growth and on their feature of molecular carriers to facilitate the translocation of drugs through brain, tumor, and cellular barriers, and finally to target glioma-specific receptors. In this contribution, the authors illustrate different molecular targets that were tested in preclinical studies (i.e., CXXR4, EGFR, AKT, VEGFR-2, MEK/ERK, integrins, MDGI, tenascin-C and neuropilin-1) and the induced effects of the interaction with specific therapeutic peptides. Of note, the authors underline that since in malignant gliomas several pathways are normally altered, better outcomes may result from combining multi-target strategies rather than interfering toward a single effector. In this context, due to their relatively reduced steric dimensions, peptides might be administered in specific combinations in order to interfere with the multitude of glioma-dysregulated pathways. Confirming this, in the last years, several preclinical studies with different types of peptides moved in this direction, providing promising results in murine models of disease and opening new perspectives for peptide applications in the treatment of high-grade brain tumors.

Gliomas are specifically characterized by the presence of hypoxic regions, especially within the tumor core, leading to an increase in vascularity. This increased vascularization is mainly driven by the expression of the major angiogenic inducer vascular endothelial growth factor (VEGF) and by the epidermal growth factor (EGF), which in turn stimulates VEGF expression. With their contribution, Nicolas and collaborators [3] analyze the regulation of VEGF axis within both hypoxia and the EGF signaling pathway in human established GBM cell lines SF-268 and U87-MG. The authors also examine the involvement of pathways downstream from EGF signaling, including the mitogen-activated protein kinase/extracellular regulated kinase (MAPK/ERK) and the phosphatidylinositol-3-kinase/RhoA/C (PI3K/RhoA/C) pathways. These in vitro molecular results allowed the authors to formulate a parsimonious model according to which hypoxia leads to an increase in VEGF expression and secretion in GBM cells, involving a dysregulated EGF receptor expression and the activation of the MAPK pathway. Hypoxia also suppresses the PI3K/Rho-GTPase pathway, potentially through an increase in the expression of RhoA GAP, StarD13 proteins. Although further effort is needed in order to elucidate the complex interplay of the molecular mechanisms involved in the hypoxic process, this contribution might underline a strictly regulated subset of molecular targets to counteract one of the main features of gliomas in future preclinical investigations. 

Particularly for gliomas, novel biomarkers associated with the progression of malignancy degrees and with poor prognosis are especially required. For this reason, the group lead by Prof. Schiffer [4] exploit the role of neuron glial antigen 2 (NG2) or chondroitin sulphate proteoglycan 4 (CSPG4) on the progression and survival of sixty-one adult gliomas and nine GBM-derived cell lines. Different studies have reported that the extracellular domain of NG2/CSPG4 protein intervenes in the regulation of the neuronal network [13], while the intracellular domain interacts with signal-regulated kinases 1 and 2 (ERK1/2) and protein kinase C-alpha (PKCα) that, in turn, regulates proliferation, migration, invasion, cytoskeletal reorganization, survival, and chemo-resistance [14]. In the examined cohort of gliomas, the authors report that NG2/CSPG4 is frequently over-expressed in IDH mutant/1p19q-codel oligodendrogliomas (59.1%) and in IDH wildtype GBMs (40%), while it is barely expressed in IDH mutant or IDH wildtype astrocytomas (14.3%). Furthermore, after immunohistochemical examinations in GBM-derived cell lines, NG2/CSPG4 expression is significantly associated with *EGFR* gene amplification and poor prognosis in the parental astrocytic tumors. Altogether, according to the authors, these results could have prognostic and therapeutic relevance, identifying NG2/CSPG4 as a promising tumor-associated antigen for future antibody-based immunotherapy in patients with malignant gliomas. 

It is becoming more and more evident that the capacity of infiltration of GBM cells into the unaffected brain tissue surrounding the tumor mass is largely responsible for tumor reoccurrence, thus reflecting on the limited efficacy of current standard-of-care treatments. In relation to this important topic, Thompson and Sontheimer [5] perform a study in large GBM datasets in parallel with human GBM cell lines and in patient-derived xenograft lines in order to shed light on the signaling axes that govern the invasive GBM behavior. The biological question raised by the authors is to determine if the grown of gliomas is associated to the presence of the neurotransmitter acetylcholine (Ach) and its receptors (AChRs), which can be both found in the growing tumor areas, and, therefore, to understand the interplay between the neurotransmitter circuit and GBM evolution. Firstly, the authors analyze RNA-Seq data from the “The Cancer Genome Atlas” (TCGA), confirming the expression of AChRs; then, they demonstrate the physiological functionality of these receptors in GBM cells by means of time-lapse calcium imaging. Following this, adopting additional in silico analyses within the “Ivy Glioblastoma Atlas Project”, they highlight that AChRs are significantly upregulated in brain regions characterized by highly infiltrated GBM cells. Further in situ evidence from the “Repository for Molecular Brain Neoplasia Data” (REMBRANDT) dataset also highlights the co-expression of choline transporters, choline acetyltransferase, and vesicular acetylcholine transporters, suggesting that GBMs express all the proteins required for ACh synthesis and release. In conclusion, this work identifies ACh as a modulator of GBM infiltration behavior and suggests that glial tumor cells may utilize the neurotransmitter as an autocrine signaling molecule to promote their tumor growth.

One of the main adverse clinical features of gliomas is the high probability of tumor recurrence, mainly mediated by a cellular subpopulation with stem cells characteristics known as glioblastoma stem-like cells (GSCs) [14]. These cells are localized in specific niches within the tumor, associated with a specific blood vessels architecture providing appropriate oxygen and energy supply [15]. Of note, these cells are reported to release high levels of extracellular adenosine, with increased levels associated in hypoxia, a microenvironment condition typical of high-grade GBM, thus enhancing tumor aggressiveness. In this regard, Niechi and collaborators [6] present an in vitro study using U87-MG astrocytoma cells subjected to direct administration of the recombinant adenosine deaminase (ADA) enzyme to induce a degradation of adenosine. This treatment resulted in a decrease in cell invasion but also tumor aggressiveness, suggesting a new therapeutic avenue for gliomas. In conclusion, the authors suggest the possibility to expand this research with in vivo studies to evaluate the infiltration capability of GSCs and to develop suitable therapeutic ADA delivery methods.

As already stated, the invasiveness of high-grade gliomas depends on their high infiltration capacity to invade the basement membranes of the surrounding brain tissues. Glioma cells are reprogrammed to have increased motility via weakened cell adhesions and through molecular mechanisms that results in a dysregulated cytoskeleton, a process overall known as epithelial–mesenchymal transition (EMT), further characterized by multiple biochemical changes that culminate in mesenchymal phenotypes [16]. This relevant topic was investigated by Takashima and co-workers [7], who performed multivariable analyses using the expression data and clinical information from glioma patients’ datasets deposited within “The Cancer Genome Atlas (TCGA)” and the “Chinese Glioma Genome Altas (CGGA)”. The authors, through a deep bioinformatics analysis, identified a specific expression signature comprising 22 genes, i.e., *DSG3*, *FN1*, *IGFBP2*, *CLDN1*, *HDAC7*, and *L1CAM*, useful for predicting the prognosis of GBM, based on the status assessment of EMT and GSCs. As a future prospective, the authors underline the potentiality of the clustered gene expression data in evaluating the survival of glioma patients, which would help to develop novel therapeutics strategies and to provide de novo marker candidates to increase the prediction of the prognosis of high-grade gliomas.

In relation to the prognosis of gliomas, the genetic characterization of glioma patients into isocitrate dehydrogenase (IDH) mutant and wildtype (IDH^mut^/IDH^wt^) sub-types has provided additional criteria in a better definition of the patients’ outcome [17]. To face this important hallmark, Dao Trong and collaborators [8], using patient-derived IDH^mut^ GSCs lines, perform a large-scale screening of 147 Food and Drug Administration (FDA)-approved anticancer compounds that are able to promote inhibition of cell growth and induce programmed cell death processes. In particular, within their in vitro analysis, the authors identify seven promising FDA-approved drugs that should be further taken into clinical investigations for the treatment of IDH^mut^ glioma sub-types. These drugs include the proteasome inhibitors bortezomib and carfilzomib, three anthracyclines (i.e., doxorubicin, daunorubicin, and epirubicin), as well the antineoplastic antibiotic plicamycin and the protein translation inhibitor omacetaxine. As a future therapeutic perspective, the authors stress the fact that the tested drugs are already FDA approved, and, therefore, they might enable faster translation into in vivo preclinical studies and subsequent clinical trials.

In the last contribution of this Special Issue, the group of Prof. Florio review the intriguing link between the cellular prion protein (PrP^C^) and gliomas, specifically in the maintenance and expansion of GSC population [9]. PrP^C^, the physiological counterpart of the pathogenic prion protein (PrP^Sc^), has become a relevant player in the oncology field. In fact, several pieces of evidence provided a pivotal role of PrP^C^ in tumorigenesis, cancer progression, acquisition of a multidrug resistance phenotype, and metastatic propagation, even in glial tumors [18]. The authors highlight that PrP^C^ in particular is overexpressed in cancer stem cells (CSCs) from different tumors, including gliomas, and that this molecular signature is predictive for poor prognosis and correlates with tumor relapses. At a cellular level, the authors illustrate the possible mechanistic contribution of PrP^C^ to multipotency, invasiveness, and tumorigenicity features of GSCs. However, due to the high number and complexity of PrP^C^ interactors and molecular pathways, future progress is required in understanding the function of PrP^C^ and of its paralogue prion Doppel protein (Dpl, [19]) within the glioma transformation process. However, this knowledge could represent a significant milestone for effective advancement in the development and implementation of PrP^C^-targeting therapeutic strategies to finally improve cancer patient management.

In conclusion, I believe that the papers collected in this Special Issue, each addressing a specific aspect of glial tumor biology, will help the scientific community to better understand the complex underlying mechanisms of glioma tumorigenesis and will help to design more effective therapeutic strategies that are able to interfere with a tumor showing a very poor prognosis to date.
